# A Habitat Model for Assessing the Impact of the Three Gorges Project on Phytophilic Spawners

**DOI:** 10.1002/ece3.72166

**Published:** 2025-09-17

**Authors:** Chenguang Xiang, Wei Huang, Cuixia Yao, Huaidong Zhou, Zhuowei Wang, Jing Wang, Pan Yang

**Affiliations:** ^1^ State Key Laboratory of Water Cycle and Water Security China Institute of Water Resources and Hydropower Research Beijing China; ^2^ Kunming Engineering Corporation Limited PowerChina Kunming China; ^3^ College of Hydrology and Water Resources Hohai University Nanjing China; ^4^ College of Water Conservancy and Environment Three Gorges University Yichang China

**Keywords:** habitat function, habitat model, sticky egg, suitability index, three gorges reservoir

## Abstract

Riverine ecosystems provide essential functional habitats for fish reproduction through dynamic hydrological regimes. However, reservoir operations fundamentally alter these natural hydrologic rhythms, creating novel selective pressures on aquatic organisms. This study investigates how dam‐induced hydrological changes affect phytophilic spawners ( *Cyprinus carpio* and *Carassius auratus* ) in the Three Gorges Reservoir—a group that has come to dominate the fish assemblage due to the shift from lotic to lentic conditions following impoundment. We developed a process‐based habitat model that integrates hydraulic parameters with an innovative water level fluctuation suitability index to quantify functional habitat loss. Our results demonstrate that reservoir drawdown regimes reduce high suitability incubation areas by 39.5% (with WUA decreasing by 34.3%), effectively compressing viable spawning microhabitats into discrete fluvial segments. The altered hydrological conditions create temporal mismatches between embryonic developmental phases and habitat stability windows, disproportionately affecting species with specialized oviposition strategies. These findings provide a basis for ecological regulation to enhance the suitability of spawning habitats.

## Graphical abstract

This study investigates how dam‐induced hydrological changes affect the reproductive strategies of sticky egg‐producing fish ( *Cyprinus carpio* and *Carassius auratus* ) in the Three Gorges Reservoir. We developed a process‐based habitat model integrating hydraulic parameters with an innovative water level fluctuation suitability index to quantify functional habitat loss.

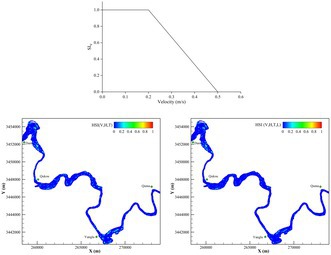

## 1 Introduction

River habitats play a critical role in aquatic ecosystems, providing essential resources and environmental conditions for the survival and reproduction of aquatic organisms (Costanza et al. 1997). However, human activities—particularly reservoir construction—have significantly altered the natural hydrological conditions of many rivers worldwide. These alterations impose novel selective pressures on aquatic organisms, especially fish species with specialized reproductive strategies such as sticky egg attachment (Cardinale et al. 2012; Dudgeon et al. 2006; Mikhailov and Kravtsova 2015). The disruption of natural water level regimes during critical incubation periods poses a severe threat to the survival of early life stages; yet, quantitative assessments of such impacts remain scarce (Kemp et al. 2011; Winfield et al. 2015; Yi et al. 2017).

Water level fluctuations in reservoirs, which can vary in amplitude, frequency, and duration, are influenced by climate‐driven changes in precipitation or by planned drawdowns (Logez et al. 2016). The dispatching operations of large reservoirs, particularly during spring and summer, cause continuous water level declines to ensure dam and downstream safety during flood seasons (Hirsch et al. 2017). However, sticky fish eggs in reservoir areas typically need to adhere to aquatic vegetation for successful hatching. A drop in water levels leads to egg desiccation and mortality in shallow littoral zones due to exposure to air (Lima et al. 2017). Addressing these ecological impacts is essential for sustainable reservoir management and requires a deeper understanding of how water level fluctuations affect fish habitats, along with the development of strategies to mitigate these effects (Chen and Olden 2017; Li et al. 2023).

In many studies, comprehensive hydrodynamic and habitat‐based methods have been adopted to evaluate the ecological status of rivers and the suitability of fish habitats (Pisaturo et al. 2017; Vilizzi 2002). Aquatic organisms exhibit a strong preference for specific ranges of hydraulic parameters (Girard et al. 2014; Macura et al. 2012). Preference curves are commonly used in ecological research at micro‐ and mesoscales to describe and assess fish habitat utilization in relation to hydraulic parameters (Parasiewicz et al. 2013; Vismara et al. 2001). However, most previous studies have focused on the suitability of velocity, water depth, sediment, water temperature, and other environmental factors (Gebrekiros 2016; Teal et al. 2018; Yi et al. 2017), while largely overlooking stage‐specific requirements—particularly the vulnerability of egg incubation to water level fluctuations. For sticky‐egg producers, successful reproduction requires not only appropriate spawning substrates but also stable hydraulic conditions throughout the embryonic development period. This study addresses a critical knowledge gap by explicitly distinguishing between spawning suitability (governed by hydraulic parameters) and hatching suitability (driven by water level stability). The decoupling of these two reproductive phases under reservoir operations represents a fundamental shift in functional habitat connectivity, which forms the core innovation of our work.

The Three Gorges Dam (TGD), the world's largest hydroelectric dam, has fundamentally altered the ecological processes of the Yangtze River system, affecting both the reservoir area and downstream habitats (Zhang et al. 2021). Most researchers have focused on the effects on protected fish downstream of the dam site, with less attention given to fish in the Three Gorges Reservoir area (Ban et al. 2019; Han et al. 2020; Zhou et al. 2014). Previous studies have primarily focused on fish that produce drifting eggs, such as the four major Chinese carps and endemic Yangtze River species (Xia et al. 2016; Xiao et al. 2022), while few have examined species that produce sticky eggs within the reservoir area. The impoundment of the Three Gorges Project has triggered a significant shift in the fish assemblage structure: drifting‐egg‐producing species (e.g., the four major Chinese carps) have retreated to the fluctuating backwater zone near the reservoir tail, while lentic‐adapted sticky‐egg producers now dominate the tributaries (> 50 species; Lin et al. 2019). These sticky eggs require specific attachment substrates for successful incubation—a need fulfilled by the extensive aquatic vegetation in the drawdown zones of reservoir tributaries. However, the spawning season in the Three Gorges Reservoir area coincides with a period of rapidly declining water levels (late April to early June) (Mu et al. 2014; Zhang et al. 2020). As a result, sticky fish eggs often fail to hatch due to desiccation from water level drops. This ecological challenge underscores the need for a more nuanced understanding of how reservoir operations disrupt the relationship between spawning habitats and fish populations.

In the present study, we combined a habitat preference index with a two‐dimensional hydrodynamic model, using carp and crucian carp as focal species to analyze the influence of TGD operations on the habitat of the Pengxi River, a tributary in the reservoir area. Specifically, we developed a novel two‐stage habitat assessment framework: (1) spawning suitability, integrating velocity and water depth preferences for egg attachment, and (2) hatching suitability, incorporating a 5‐day water level fluctuation index to quantify developmental stability requirements. This approach provides the first quantitative tool to disentangle reservoir impacts on the sequential reproductive phases of sticky‐egg‐producing fishes.

## 2 Materials and Methods

### 2.1 Study Area and Fish Data

**FIGURE 1:**
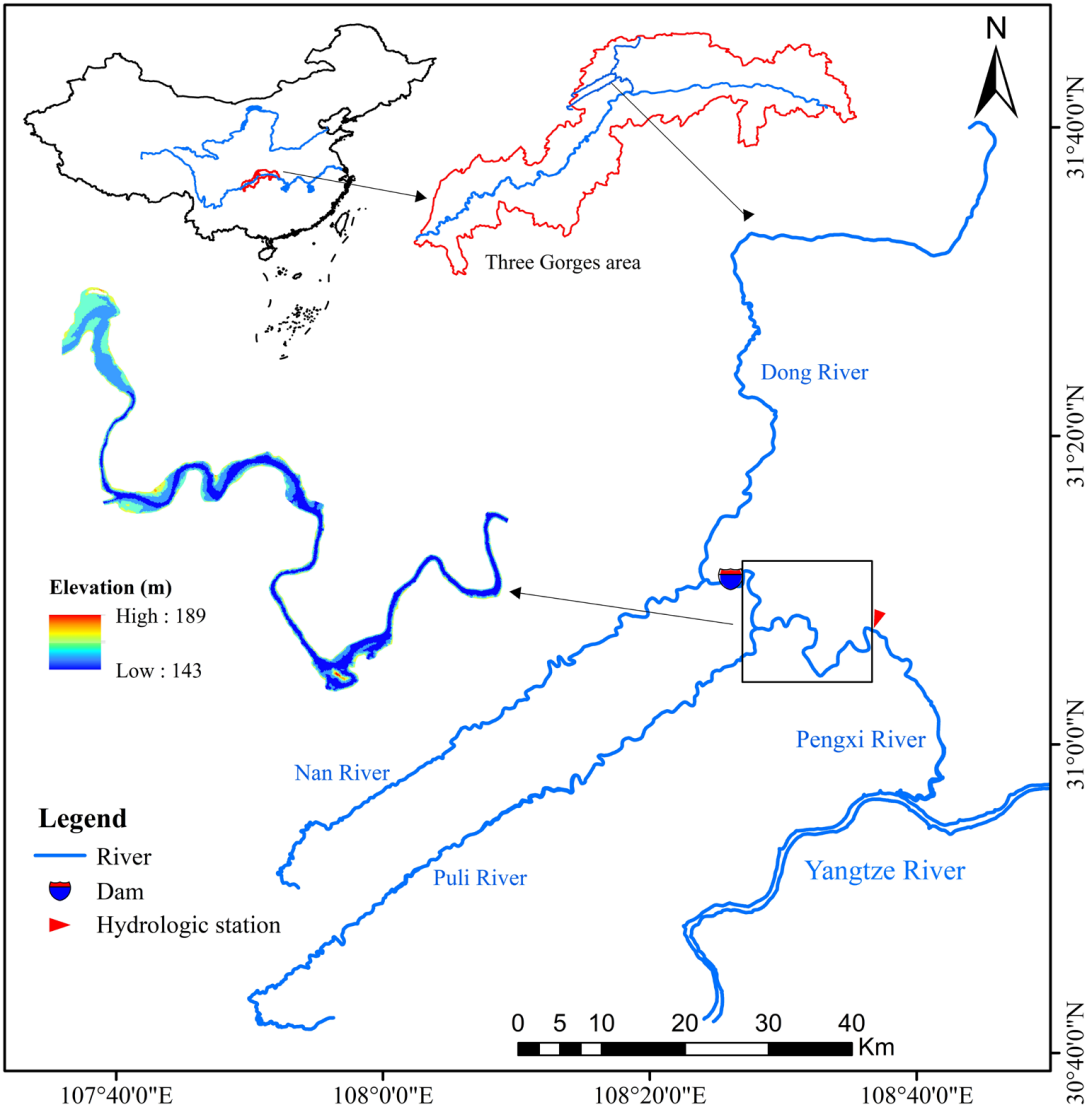
Map of the study area.

A large area of herbaceous vegetation exists in the water–land ecotone of tributaries in the Three Gorges Reservoir area, providing a wide range of attachment substrates for sticky‐egg‐producing fish such as carp and crucian carp. In contrast, the gravel shoals at the backwater ends of tributaries and in the mainstem provide suitable spawning grounds for species that lay eggs on gravel (Que et al. 2018). The Pengxi River, a tributary of the Three Gorges Reservoir, offers spawning grounds for sticky‐egg producers and was therefore selected as the research area. Located at the eastern edge of the Sichuan Basin (107°56′–108°54′ E and 30°49′–31°42′ N), the Pengxi River has a drainage area of 5173 km^2^ and a total length of 182.4 km. It is the largest tributary on the north bank of the Three Gorges Reservoir area (Sun et al. 2021). The study area is illustrated in Figure 1.

Fish that produce sticky eggs in the static and slow‐flowing habitats of the backwater area in the reservoir include *Cyprinus carpio* Linnaeus, *Carassius auratus* , *Hyporhamphus intermedius* , *Hemibarbus* o*bscur*u*s* Bleeker, *Misgurnus anguillicaudatus* , *Paramisgurnus dabryanus* Sauvage, *Sinibrama wui* , and *Pseudorasbora parva* (Yang et al. 2012). Protection value, resource abundance, and sensitivity to fluctuations in reservoir water levels were taken into account when selecting the target species. Compared with other sticky‐egg‐producing species, carp ( *Cyprinus carpio* Linnaeus) and crucian carp ( *Carassius auratus* ) are the most abundant in the Three Gorges Reservoir area. Therefore, carp and crucian carp were chosen as the focal species for these targets.

### 2.2 Methods

#### 2.2.1 Hydrodynamic Model

A hydrodynamic model of the Pengxi River, a tributary of the Three Gorges Reservoir, was developed using the HD module of the MIKE21 FM model to simulate the hydrodynamic characteristics of the reservoir's tributary area. The MIKE21 FM two‐dimensional unsteady flow module is based on the Reynolds‐averaged stress equations for a two‐dimensional incompressible fluid, following the Boussinesq approximation and the hydrostatic pressure assumption (Ahn et al. 2019).

#### 2.2.2 Habitat Model

The habitat suitability model (HSM) has been widely used to assess habitat availability for aquatic organisms, such as fish, and to predict the effects of human activities on aquatic ecosystems (Bovee et al. 1998). The HSM describes the relationship between the specific behavioral characteristics of aquatic organisms and their surrounding habitats. The habitat suitability index (HSI) is used to quantify the degree of fish preference for various abiotic variables (Boavida et al. 2014). A habitat suitability curve (HSC) was constructed after determining the biological requirements of each target species.

From March to June 2019, our research group conducted a survey on early life stage fish resources in the Pengxi River, covering the section from the Hanfeng Lake Regulating Dam to the Yanglu River. Fish larvae and egg sampling were conducted using hand nets and trawl nets deployed across various substrates within the littoral zone, while corresponding environmental parameters were recorded. The survey revealed that fish with sticky eggs preferentially occupied nearshore habitats characterized by low flow velocities, with the annual spawning peak occurring between April and May. Fish eggs were primarily found adhered to aquatic vegetation in the littoral zone, where flow velocities ranged from 0 to 0.5 m/s, with most areas exhibiting velocities below 0.2 m/s.

**FIGURE 2:**
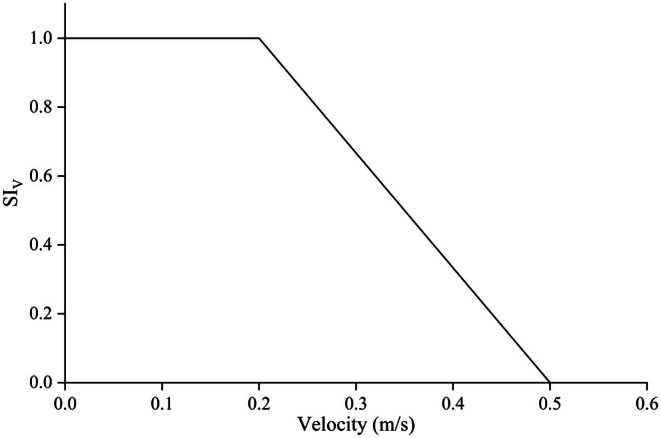
Habitat suitability curve based on velocity.

**FIGURE 3:**
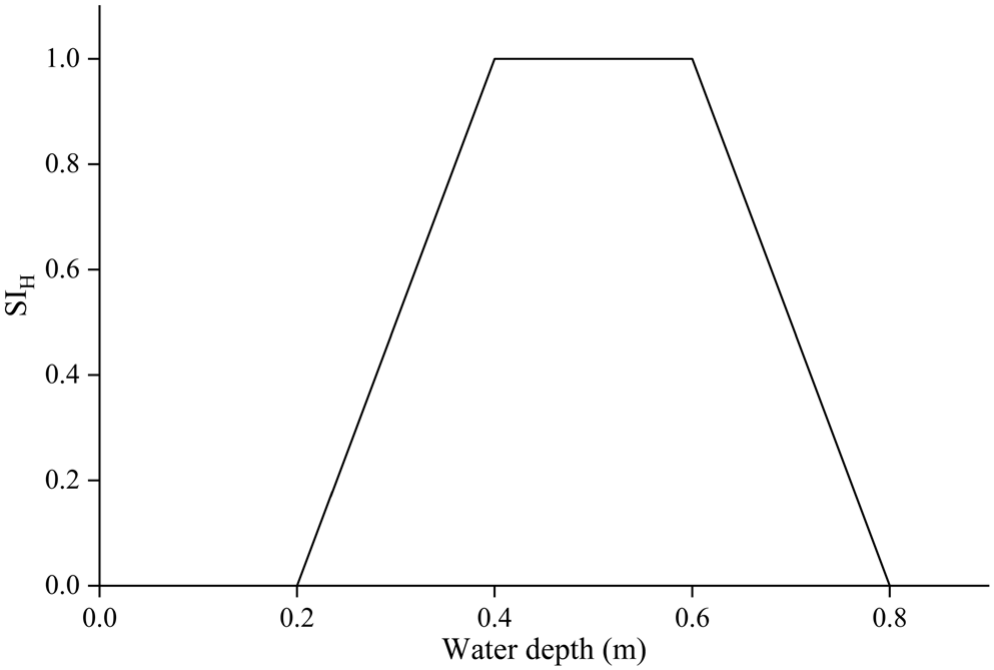
Habitat suitability curve based on water depth.

**FIGURE 4:**
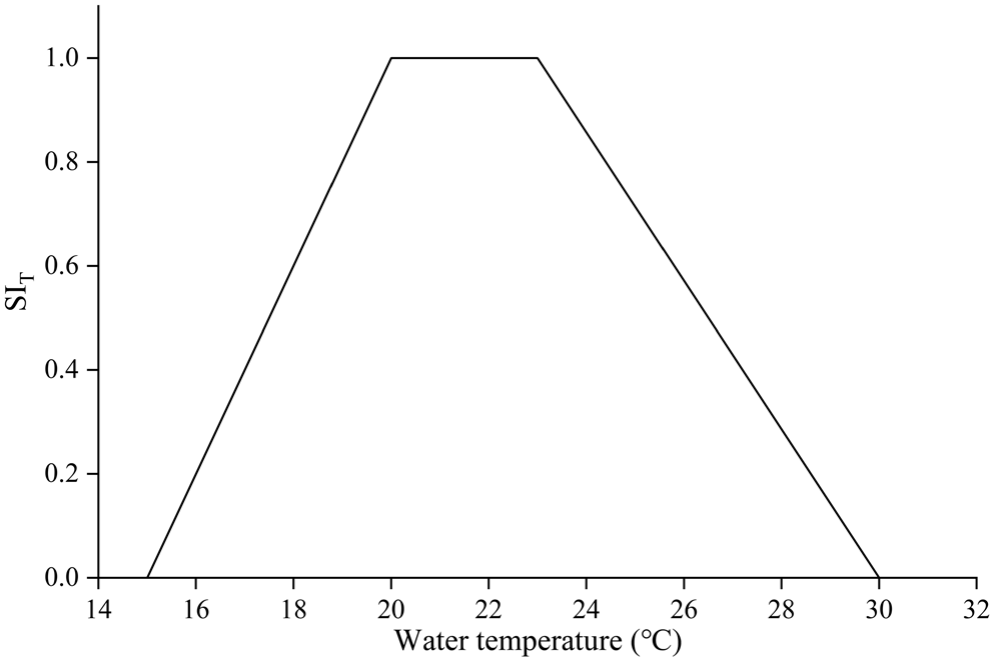
Habitat suitability curve based on water temperature.

The water temperature during the collection of cyprinid eggs (carp and crucian carp) ranged from 15°C to 30°C, with a mean of 20.2°C. Eggs were most frequently collected at temperatures between 20°C and 23°C, accounting for 68.1% of the total samples. Collections below 20°C and above 23°C accounted for 15.9% and 16.0% of the total, respectively. Adhered eggs were observed at water depths ranging from 0.2 to 0.8 m. The highest frequency of egg attachment occurred at depths of 0.4–0.5 m, constituting 33.1% of observations, followed by depths of 0.5–0.6 m (25.2%) and 0.3 to 0.4 m. No egg attachment was observed at depths exceeding 80 cm. This study defined the central range encompassing approximately 60% of observations as the most favorable range for the target species. Consequently, the optimal ranges for flow velocity, water depth, and water temperature were determined to be 0–0.2 m/s, 0.4–0.6 m, and 20°C–23°C, respectively. A suitability index (SI) value of 1 was assigned to these optimal ranges, while the upper and lower limits of the environmental variable ranges were assigned SI values of 0. The suitability curves for flow velocity, water temperature, and water depth are presented in Figures 2, 3, 4.

In situ observation trials demonstrate that, under suitable temperature conditions, the incubation period for carp eggs ranges from 42 to 51 h, while for crucian carp it is approximately 40–49 h. Collectively, the spawning and hatching of carp and crucian carp typically require approximately 2 days. Furthermore, cyprinid larvae only attain a measurable level of free‐swimming ability 3–4 days post‐hatching. The complete process from hatching to achieving sustained free‐swimming generally requires more than 5 days. Consequently, a critical window of at least 5 days—from egg deposition (spawning) to the attainment of free‐swimming capability—exists, during which egg and larval survival must be ensured.

**FIGURE 5:**
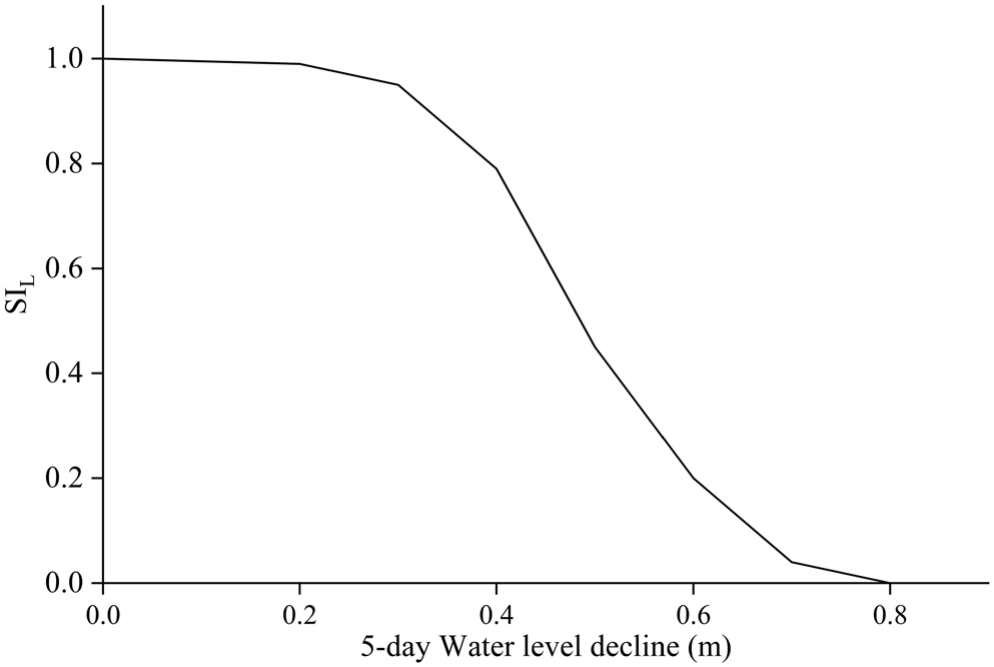
Habitat suitability curve based on 5‐day water level drop.

Based on the frequency distribution table of spawning water depths for carp ( *Cyprinus carpio* ) and crucian carp ( *Carassius auratus* ) (Table 1), the varying distribution of eggs at different water depths results in different proportions of eggs being adversely affected depending on the magnitude of water level decline. When the water level fluctuation is 0 on the fifth day, no fish eggs are affected, and the SI value is 1. When the total water level drops to less than 0.2 m by the fifth day, only 0.6% of fish eggs are affected, and the SI value is 0.994. When the water level drops by more than 0.8 m, habitat suitability is at its lowest, and the SI value decreases to 0. The relationship between water level fluctuations and habitat suitability shows a linear trend, with varying proportions of egg loss corresponding to different magnitudes of water level decline. Therefore, the suitability curve for water level fluctuation is shown in Figure 5.

**TABLE 1:** Distribution frequency of fish eggs of *Cyprinus carpio* and *Carassius auratus* at different water depths.

Water depth range (m)	Frequency	Proportion (%)
0.0–0.2	1	0.6%
0.2–0.3	7	4.3%
0.3–0.4	27	16.6%
0.4–0.5	54	33.1%
0.5–0.6	41	25.2%
0.6–0.7	26	16.0%
0.7–0.8	7	4.3%

To further quantify habitat requirements, we developed a habitat suitability equation based on the spawning and hatching characteristics of carp and crucian carp. The spawning suitability equation reflects the preferences of carp and crucian carp for flow velocity, water depth, and temperature during the spawning period. The hatching suitability equation incorporates the suitability index of the 5‐day water level drop, which reflects the influence of dam operation schemes on the hatching success of carp and crucian carp eggs. When the habitat suitability of any factor is zero, the geometric mean habitat suitability across all factors is also zero. Therefore, the key factors affecting habitat suitability can be calculated using the geometric mean method:

(1) $$\begin{array}{l} \text{Spawning suitability}:{\mathrm{HSI}}_{i}={\mathrm{SI}}_{V}\times {\mathrm{SI}}_{H}\times {\mathrm{SI}}_{T}\;\\ \text{Hatching suitability}:{\mathrm{HSI}}_{i}={\mathrm{SI}}_{V}\times {\mathrm{SI}}_{H}\times {\mathrm{SI}}_{T}\times {\mathrm{SI}}_{L} \end{array}$$

where SI_V_, SI_H_, SI_T_, and SI_L_ represent the appropriate values of flow velocity, water depth, temperature, and 5‐day water level decline of unit I, respectively, and HSI_i_ is the HSI value of each unit i. This corresponds to five habitat suitability categories (Boavida et al. 2018), that is, very low (0–0.2), low (0.2–0.4), medium (0.4–0.6), high (0.6–0.8), and very high (0.8–1.0).

The habitat simulation output can be expressed using the weighted usable area (WUA). These variables were defined using the following equation (Xiao et al. 2022):

(2) $$\mathrm{WUA}=\sum_{i=1}^{n}\left(A_{i}\times {\mathrm{HSI}}_{i}\right)$$

where *A*_i_ is the area of the calculation unit I.

#### 2.2.3 Model Setup

All models were coupled. The initial and boundary conditions of the hydrodynamic habitat model were defined as follows: the inlet was assigned a flow boundary condition, and the outlet was controlled using specified boundary conditions. A zero‐gradient condition was applied to velocity, and a solid boundary condition was assigned to the side boundaries. The simulation period spanned April to May, coinciding with the peak spawning season for carp and crucian carp, and utilized typical data from 2019 to 2021, following the construction of the Three Gorges Reservoir. After running the hydrodynamic model, the fish habitat preference curves were imported to generate the spatial distribution of habitat suitability factors.

## 3 Results

### 3.1 Habitat Model Validation

**FIGURE 6:**
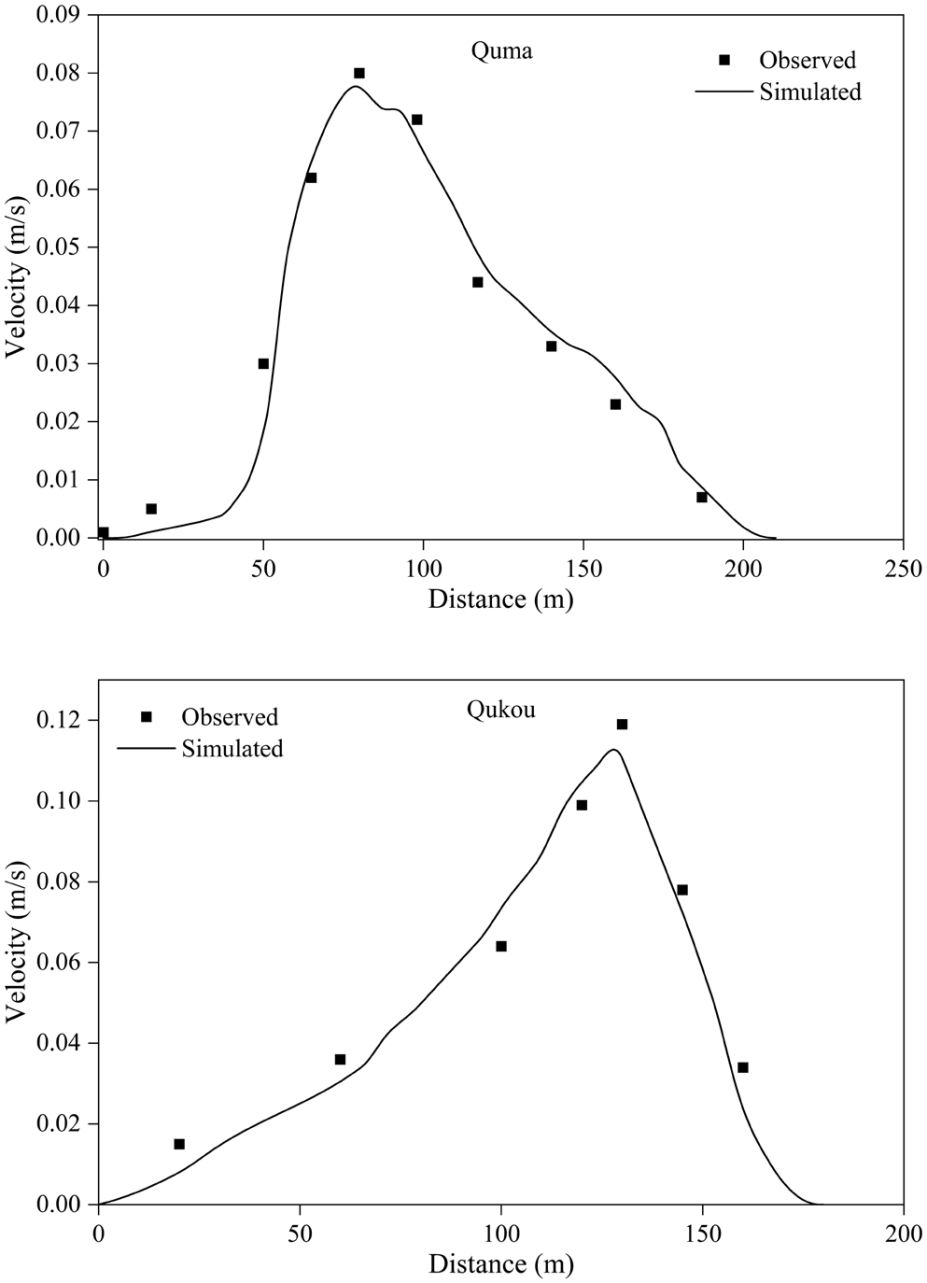
Verification of the flow velocity in the Quma and Qukou sections.

The velocity data from the Quma and Qukou observation sections on May 6, 2019, were used for comparison and verification, and the roughness was calibrated accordingly. The simulation results after calibration are shown in Figure 6. The correlation coefficients between the simulated and observed values were 0.91 and 0.89, indicating that the simulation results are reliable and that the model is suitable for simulating the Pengxi River.

**FIGURE 7:**
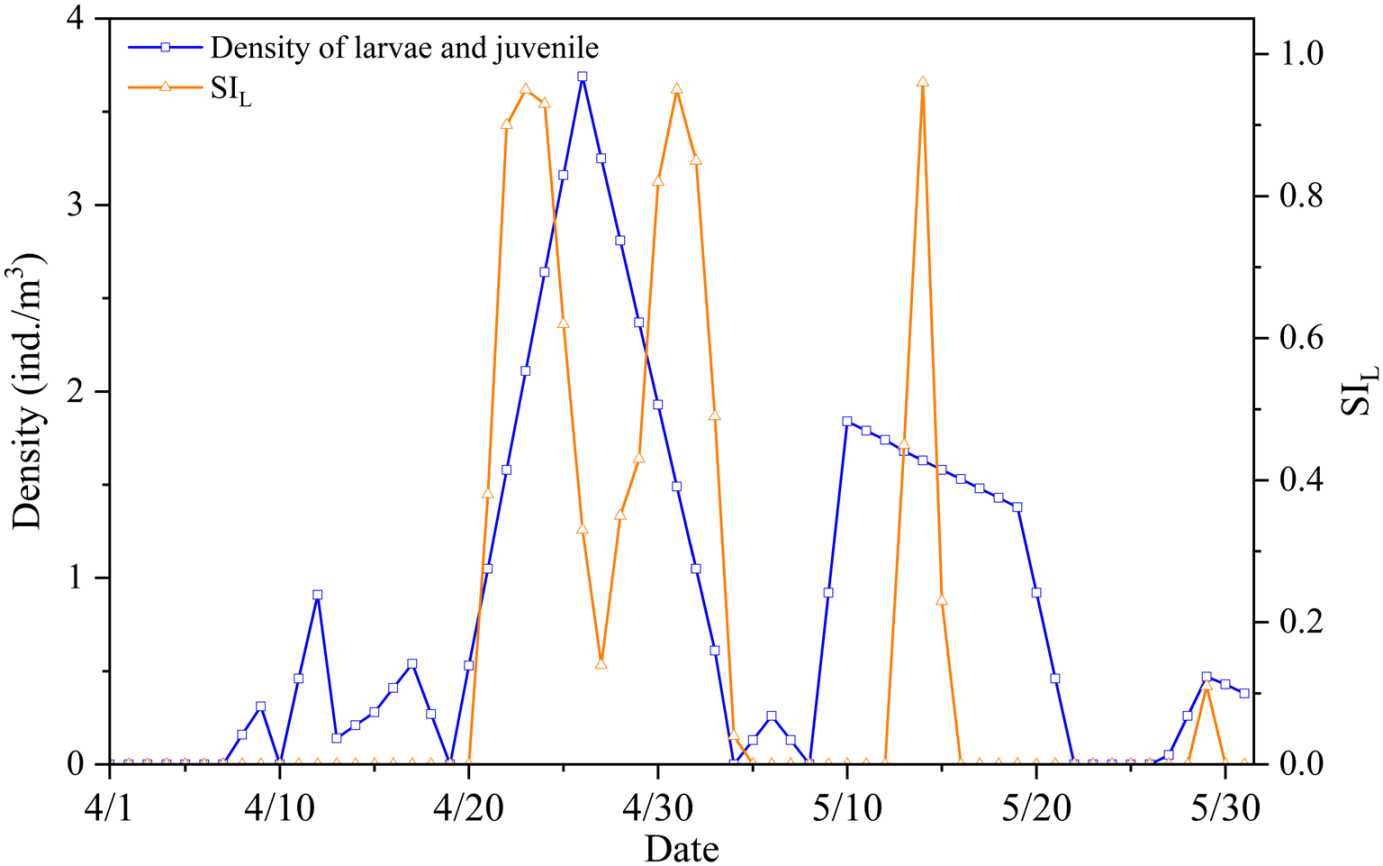
Comparison of daily density of larvae and juveniles with SI_L_.

Based on the number of fish larvae and fry collected during the field investigation from April to May 2019, the suitability index for water level drop was verified. Figure 7 shows the relationship between the density of monitored larvae and the calculated suitability index (the collected larvae were generally produced 5 days earlier, so the SI_L_ time coordinate was shifted 5 days earlier). The results show that the daily density of larvae and fry is positively correlated with SI_L_, with a Pearson correlation coefficient of 0.61 (*p* < 0.05). The peak period of larvae and fry coincides with the high values of SI_L_. These findings indicate that the habitat suitability curve proposed in this study effectively reflects the hatching preferences of phytophilic spawners and predicts potential hatching sites.

In addition, monitoring data show that from April 12 to 16, 2022, the average water level in the Three Gorges Reservoir area decreased by 0.16 m/day, during which 70 million sticky eggs were produced. From April 20 to 27, the average water level dropped by 0.12 m/day, during which 41 million sticky eggs were produced. These empirical results confirm the inverse relationship between drawdown intensity and reproductive success, validating our water level fluctuation suitability index (Figure 5).

### 3.2 Effect of Water‐Level Change on Egg Hatching

**FIGURE 8:**
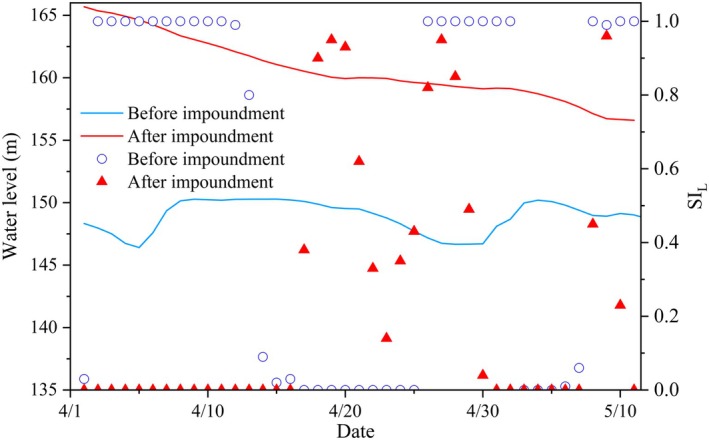
Comparison of SI_L_ before and after TGD operation.

Figure 8 shows a comparison of the water‐level change process and the suitability index of water‐level decline at Xiaojiang Station in the Pengxi River from April to May, before and after impoundment. Before the operation of the Three Gorges Dam (TGD), the water level of the Pengxi River was generally stable, with occasional significant fluctuations caused by rainfall. Following the impoundment of the Three Gorges Reservoir, April marks the period of slow decline in the reservoir's water level, with an average daily decline of −0.22 m/day. During this period, the water level remains relatively stable, with minimal impact on the hatching activities of carp, crucian carp, and other fish species. However, May represents the period of the fastest water level decline, with an average daily drop of −0.32 m/day and a maximum daily drop of −0.61 m/day. Within just 1–2 days, the water level can fall below the optimal depth range required for carp and crucian carp to lay eggs. This creates a significant threat to the survival of fish eggs, which are prone to dehydration and mortality due to exposure.

Under natural conditions, the average Suitability Index of water level fluctuation (SI_L_) for carp and crucian carp habitats was 0.57 in April and 0.55 in May, showing only a slight difference. Under these conditions, the spawning habitats exhibit high hatching suitability, and water level fluctuations have a relatively minor influence on egg survival. However, this situation changes markedly after impoundment. In April, the average SI_L_ dropped to 0.27, and in May, it plummeted to just 0.05. These findings indicate that the risk to carp and crucian carp hatching increases substantially in April and May after impoundment. In particular, the rapid decline in water levels in May has an increasingly severe adverse effect on fish reproduction.

In terms of total water level decline, there were many periods before impoundment when the 5‐day drop was less than 0.8 m, meeting the suitable conditions for egg survival. After impoundment, however, only a few slow‐decline periods met the condition where the 5‐day drop was less than 0.8 m. This further emphasizes the negative impact of post‐impoundment water level fluctuations on phytophilic spawners. Before the impoundment of the Three Gorges, SI_L_ values in the Pengxi River fluctuated due to rainfall. Although periods of low SI_L_ occasionally occurred, they were often followed by a rise in water level the next day, preventing sustained low SI_L_ periods. In contrast, after impoundment, the rapid decline of water levels during the spawning season negatively affects sticky egg survival in the Pengxi River and increases the uncertainty of fish reproduction.

### 3.3 Simulation of Changes in Habitat Environmental Factors

**FIGURE 9:**
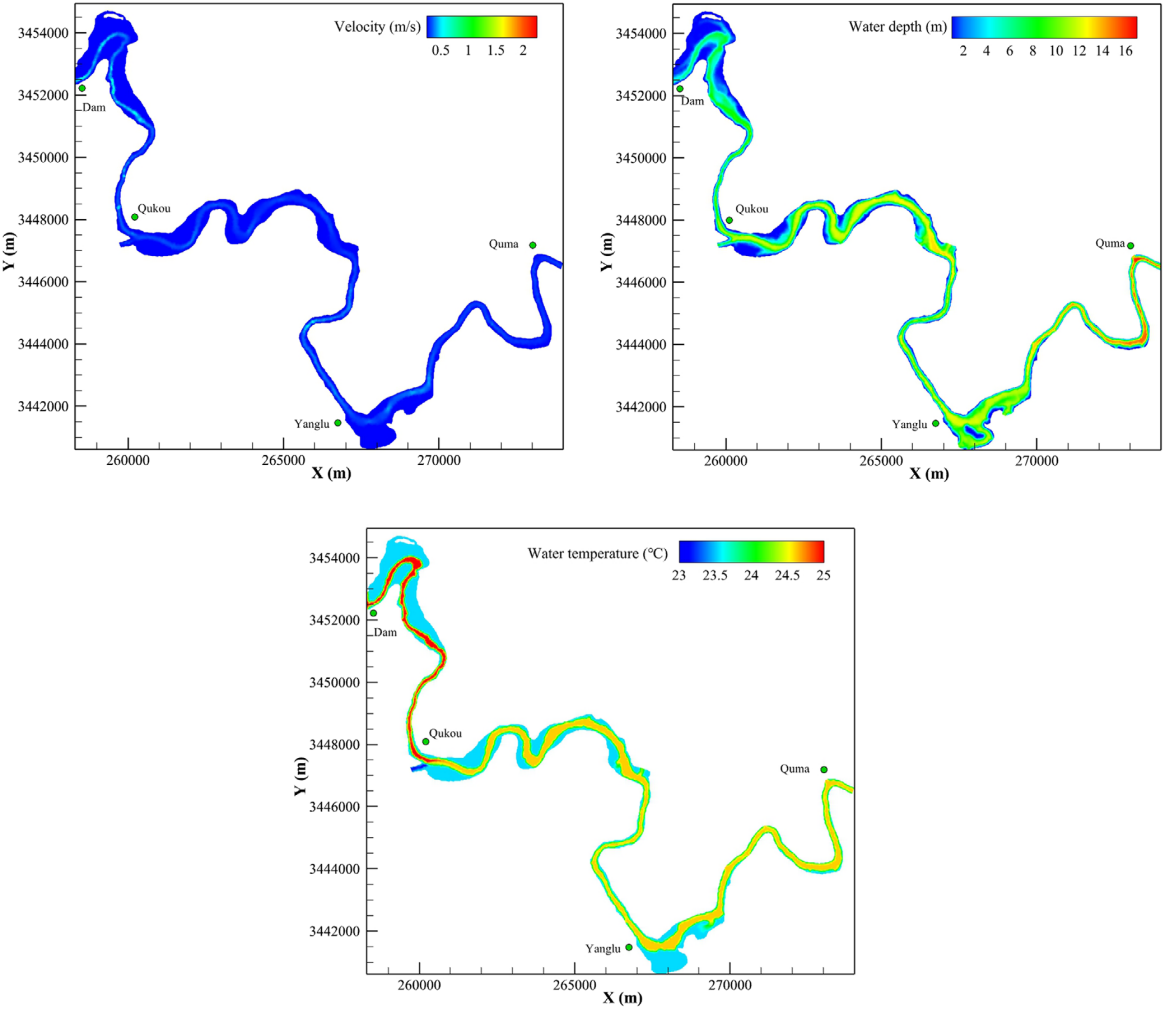
Simulated velocity, water depth, and temperature after construction of the TGD.

Taking the simulation results from May 2019 as an example, the simulated distributions of flow velocity, water depth, and water temperature in the study area are shown in Figure 9. The coordinate system used in Figure 9 is a projected coordinate system, with the X and Y axes measured in meters. Analysis of velocity changes shows that flow velocities in the Pengxi River ranged from 0.0 to 1.4 m/s from April to May following the impoundment of the Three Gorges Dam. In some areas, velocities exceeded 1.0 m/s. The flow velocity in the center of the river was slightly higher, at around 0.3 m/s, while in other areas, it remained below 0.2 m/s. After impoundment, the reduced flow velocities are suitable for the survival of carp, crucian carp, and other still‐water‐adapted species. Overall, changes in flow velocity have a positive effect on phytophilic spawners, with a large number of suitable spawning areas present in the Pengxi River.

Analysis of water depth shows that during the same period, water depths in the study area ranged from 0.0 m to 14.5 m, with depth generally increasing from upstream to downstream. The depths suitable for carp and crucian carp spawning are mainly located along both banks or in shallow areas. The river section from the dam to the canal mouth provides appropriate water depths for spawning and breeding. However, because carp and crucian carp prefer to lay eggs on aquatic vegetation along the shoreline, the decline in water level due to the Three Gorges Dam makes it difficult for water to submerge these plants, increasing the risk of egg desiccation and hatching failure.

The Pengxi River exhibits pronounced spatial heterogeneity and hydrological regulation effects in its water temperature distribution. Upstream sections show significantly higher temperatures than downstream ones, while the core reproductive zone (critical for spawning and incubation) maintains a thermally stable environment of 23°C–25°C. Within the Qukou‐to‐Yanglu River segment, temperatures reached 24.5°C ± 0.4°C—fully encompassing the optimal physiological range for carp and crucian carp—indicating high habitat suitability. Specific statistical analysis of water temperature changes from April to May reveals a noticeable gap between natural upstream conditions (range: 16.2°C–29.2°C; average: 22.9°C) and downstream areas regulated by the Three Gorges Reservoir (range: 13.7°C–26.7°C; average: 20.6°C). The mean temperature difference of 2.3°C is attributable to the cooling effect of reservoir backwater, which may delay the attainment of spawning threshold temperatures in downstream reaches by several days compared to natural flow conditions.

**FIGURE 10:**
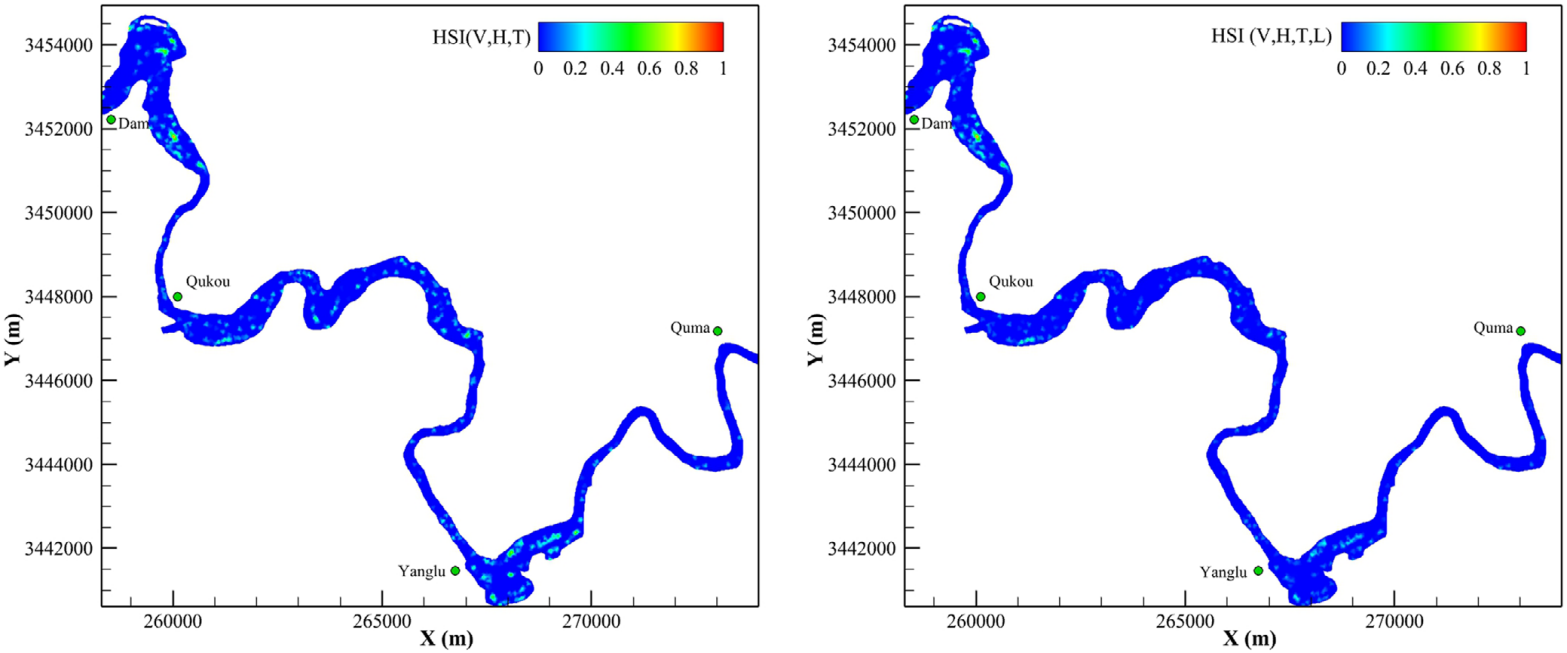
Habitat suitability distribution for spawning and hatching.

Figure 10 shows a comparison between the SI values of the study area before and after incorporating the suitability index for water level fluctuation, representing suitability for spawning and hatching, respectively. The results indicate that the proportion of very low suitability areas is the highest across the entire study area, exceeding 90%, followed by areas of very high, low, medium, and high suitability. The spawning suitability distribution map reveals many areas suitable for carp and crucian carp spawning, particularly in the Dam–Qukou and Yanglu reaches. However, the hatching suitability map indicates a substantial reduction in habitat suitability due to the decline in water levels caused by the Three Gorges Dam, with a marked decrease in the area of high suitability (HSI > 0.6). Among the affected zones, the Yanglu reach shows the most significant decline, with a considerable reduction in suitable hatching habitat. The hatching of sticky fish eggs has been severely impacted, and only a few areas suitable for the hatching of carp and crucian carp eggs remain within the reach below the dam. This finding aligns with field investigations of spawning grounds conducted after impoundment (Huang et al. 2021), confirming the reliability of the habitat model developed in this study.

Analysis of the mean spawning and hatching habitat suitability from April to May during 2019–2021 (Table 2) reveals a highly fragmented gradient in reproductive habitat suitability within the Pengxi River. The results show that 97.77% ± 0.11% of the total area was classified as having extremely low suitability (HSI = 0–0.2), while high suitability zones (HSI > 0.6) accounted for less than 1.5% of the area. Premium spawning and hatching habitats (HSI > 0.6) comprised only 0.37%–0.86% of the total area, highlighting the critical dependence of reproductive success on limited microhabitats. The high suitability area during the hatching phase (0.98%–1.10%) was consistently smaller than during the spawning phase (1.32%–1.37%), indicating a measurable impact of water‐level drawdowns, which reduced the average high suitability area by 39.5%. On average, the very high and high suitability areas for hatching sticky fish eggs decreased by 41.22% and 36.83%, respectively, while the medium, low, and very low suitability areas increased by 25.16%, 23.84%, and 0.11%, respectively. These results demonstrate that declining water levels lead to a continuous reduction in habitat area suitable for hatching carp and crucian carp eggs, and that dam operations intensify the fragmentation of fish habitats.

**TABLE 2:** Proportion of habitat area with different suitability standards.

Suitability area	Very low	Low	Medium	High	Very high
(%)	(0–0.2)	(0.2–0.4)	(0.4–0.6)	(0.6–0.8)	(0.8–1)
2019					
Spawning	97.78%	0.46%	0.40%	0.51%	0.86%
Hatching	97.87%	0.61%	0.53%	0.38%	0.61%
2020					
Spawning	97.67%	0.48%	0.44%	0.59%	0.83%
Hatching	97.80%	0.62%	0.59%	0.40%	0.59%
2021					
Spawning	97.86%	0.43%	0.39%	0.48%	0.84%
Hatching	97.96%	0.56%	0.52%	0.37%	0.59%
Average					
Spawning	97.77%	0.45%	0.41%	0.53%	0.84%
Hatching	97.88%	0.60%	0.54%	0.39%	0.60%
Amplitude of variation	0.11%	23.84%	25.16%	−36.83%	−41.22%

### 3.4 Habitat WUA Change Affected by the Operation of the Three Gorges

**FIGURE 11:**
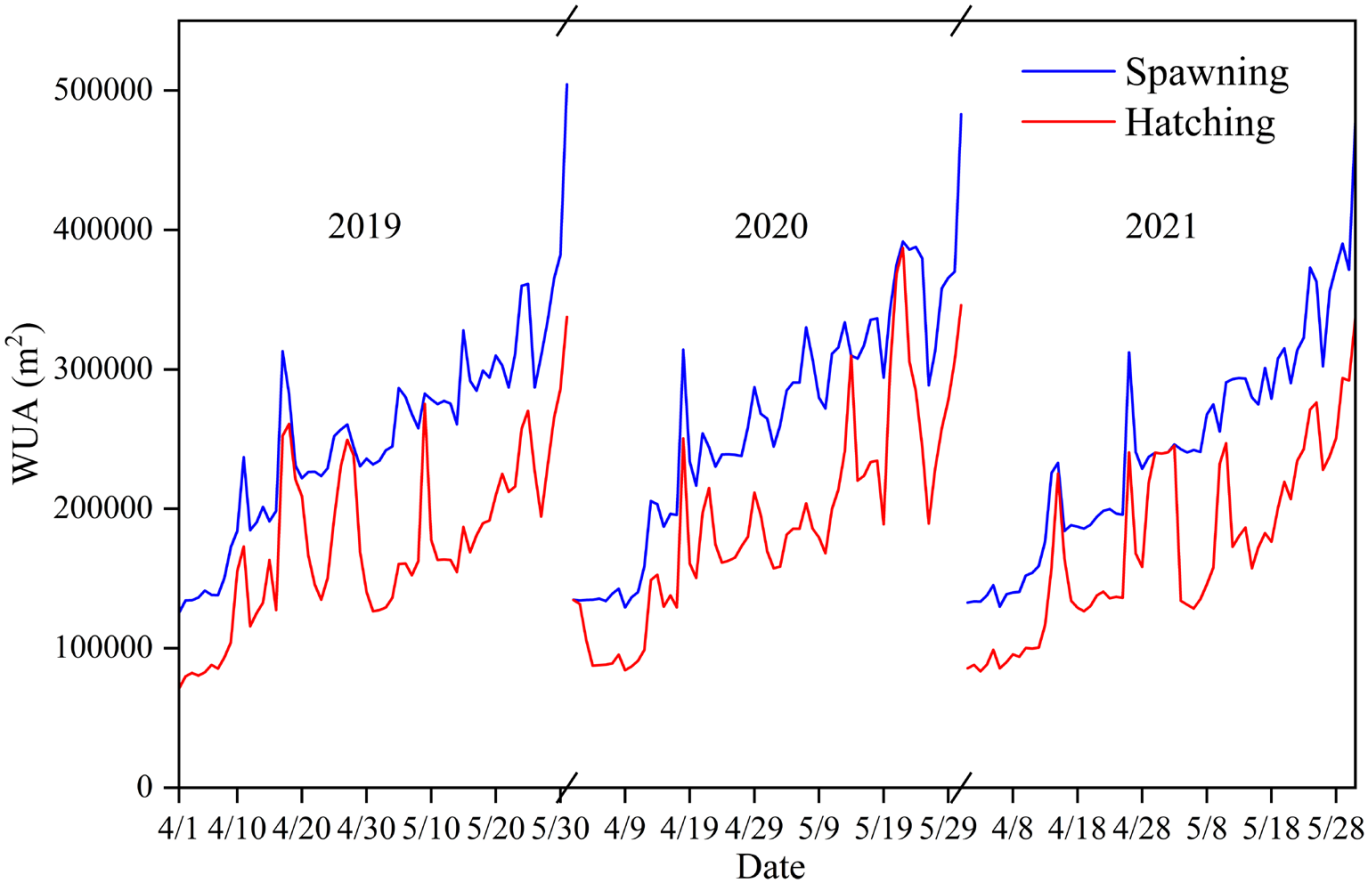
Comparison of WUA between spawning and hatching.

Figure 11 illustrates the similar temporal dynamic of the weighted usable area (WUA) for both spawning and hatching. Beginning in April, the weighted usable area of spawning and hatching presents a fluctuating upward trend, and the weighted usable area in May was higher than that in April. The primary reason is that as the water level in the Three Gorges Reservoir area continues to decline, its influence on the upstream backwater region gradually diminishes, and gradually returns to its natural flow pattern, thereby improving habitat suitability. Notably, at certain time points, the hatching WUA closely approaches the spawning WUA, indicating minimal water level drop during periods, which is beneficial to the smooth hatching of sticky fish eggs. However, hatching WUA (annual average of 166,000 m^2^) is systematically lower than spawning WUA (annual average of 253,000 m^2^), revealing a mismatch described as “high spawning potential‐low hatching realization”.

From 2019 to 2021, the hatching WUA decreased by 34.1%, 34.0%, and 34.6%, respectively, compared to the spawning WUA, with an average reduction of 34.3% of sticky eggs at risk of hatching failure due to the drop in water level. Among them, on May 31st, 2019, the most typical single‐day incubation WUA dropped from 504,000 to 338,000 m^2^ (−33.1%), directly related to a sudden water level drop exceeding 0.8 m/day, revealing that the hydrological pulse exceeded the ecological critical threshold (0.16 m/day), causing habitat damage. The suitable hatching area in April and May decreased by 77,000 and 96,000 m^2^, respectively, compared to the spawning area, indicating that the risk of the rapid drop of water level in May was higher than in April. This reduction in WUAs is directly linked to the drop in reservoir water levels during the falling stage of TGD operation, indicating that the reservoir operation scheme affected the hydrodynamic conditions of carp and crucian carp spawning during this period.

The results of this study are useful for formulating effective ecological regulation strategies to improve fish habitats in the reservoir area. Following the impoundment of the Three Gorges, water temperatures generally rose to levels suitable for carp and crucian carp spawning between April and May. When water temperatures reach approximately 20°C, ecological flow regulation can be implemented. It is recommended to conduct at least two ecological dispatches, each lasting no less than 5 days. During each ecological operation, the daily water level decline in the Three Gorges Reservoir should be maintained at approximately 0.16 m, with the total water level drop kept below 0.8 m.

## 4 Discussion

Our findings elucidate a critical mechanism by which dam operations disrupt fish reproductive strategies through stage‐specific hydraulic mismatches. Traditional habitat models primarily focus on spawning requirements, but our distinction between spawning and hatching suitability reveals that reservoir drawdowns disproportionately affect the latter phase (Gao et al. 2010; Mao et al. 2023; Zheng et al. 2021). The 39.5% reduction in high suitability incubation areas (compared to a 34.3% decline in WUA for spawning zones) demonstrates that successful egg attachment does not guarantee embryonic survival under conditions of rapid water level decline. This mechanistic decoupling explains why some restored spawning grounds fail to support population recovery. Understanding the habitat suitability response of sticky‐egg‐producing fish, such as carp and crucian carp, to hydrodynamic changes after the impoundment of the Three Gorges Reservoir is therefore of great importance.

The sharp decline in the suitability index for water level fluctuation (pre‐dam: 0.56 vs. post‐dam: 0.16) creates substantial ecological risk during critical hatching windows. While initial post‐impoundment conditions favored macrophyte colonization in newly formed littoral zones (Li et al. 2012; Quan et al. 2021; Yang et al. 2021), subsequent rapid drawdowns (> 0.6 m/day) disrupted the co‐adapted timing between egg deposition and hydraulic stability. This decoupling highlights the mismatch between the peak breeding period of carp and crucian carp (April–May) and the reservoir dispatching hydrograph, compromising the functionality of their spawning habitats. According to the reservoir operation scheme, ecological flow regulation can be effectively implemented during the descending stage by limiting the daily water level decline to below 0.16 m/day, which allows sufficient time for the hatching of sticky fish eggs (Li and Zhou 2022).

The impoundment of the Three Gorges has reconfigured the hydraulic conditions of Pengxi River tributaries, creating locally favorable environments for adhesive‐egg‐producing fish. Post‐dam flow velocity homogenization (Figure 7) renders much of the study area optimal for carp and crucian carp spawning (0–0.2 m/s), particularly along vegetated littoral zones where water depth consistently ranged between 0.4 and 0.6 m, depths accounting for 58.3% of observed egg attachment sites (Table 1). A pronounced longitudinal temperature differential emerged, with the upper reaches (22.9°C ± 3.5°C in April–May) warming more quickly than reservoir‐regulated lower reaches (20.6°C ± 2.8°C). Meanwhile, the mid‐river “thermal core” (24.5°C ± 0.4°C between Qukou and Yanglu) maintained optimal temperatures for embryo development (17°C–25°C). According to the current operational practices of the Three Gorges Reservoir, ecological operations can be carried out for carp and crucian carp once the water temperature reaches 17°C. The water temperature in the upper reaches of the Pengxi River reached 17°C on April 4, while the lower reaches reached 17°C on April 9, potentially delaying downstream spawning activity by approximately 5 days (Figure 9).

Methodologically, the suitability index of water level fluctuation advances functional habitat modeling by quantifying stage‐specific environmental stressors during fish development. In our model, traditional hydraulic parameters (velocity and depth) describe the suitability of spawning habitats, while the water level fluctuation suitability index quantifies hatching suitability. The dispatching operations of the Three Gorges Reservoir have reduced the area suitable for hatching to approximately 65.7% of the area suitable for spawning (Figure 11). The findings of our study align with fish resource surveys in tributaries of the reservoir area, confirming that the habitat model effectively predicts the likelihood of egg‐laying in the region (Huang et al. 2021). These results underscore the necessity of incorporating life‐history‐specific hydraulic conditions when assessing the impacts of anthropogenic activities on fish species.

These changes highlight the importance of integrating hydrological management with ecological considerations to reduce the impact of reservoir operations on reproductive success and the long‐term sustainability of species. The decline in water level in the Three Gorges alters habitats, primarily threatening the key incubation stage of egg‐adhering ovipositors. However, the impact extends beyond this stage. Rapid water level decline may also endanger the survival of early larvae by eliminating shallow‐water nursery habitats and increasing predation risk, and may disrupt adult oviposition behavior by altering the accessibility and stability of preferred spawning sediments (Refsnider and Janzen 2010). Additionally, fluctuations in water levels can impact benthic and plankton communities, indirectly affecting food resources across all life stages.

Importantly, not all lithophilic/demersal spawners respond equally to the hydrological alterations quantified in our model. While our study focused on the abundant carp and crucian carp, the diverse assemblage of adhesive‐egg producers in the TGR tributaries (> 50 species) exhibits variations in life‐history traits that shape their vulnerability. For example, species such as *Rhodeus sinensis* Günther may have slightly different spawning phenology; if their peak spawning coincides with the steepest drawdown period (e.g., May; Figure 8), they could face even higher embryonic mortality risks than carp and crucian carp. Similarly, species with longer egg developmental durations (e.g., exceeding the 2 days observed in carp and crucian carp, as noted in Section 2.2.2) require extended periods of stable water levels. Therefore, they are more sensitive to cumulative drops within the critical 5‐day window. Variations in preferred spawning depth (as shown by the depth distribution in Table 1 for carp and crucian carp) also influence exposure risk; species utilizing shallower zones (e.g., 0.2–0.3 m) would be impacted earlier and more severely by a given drawdown magnitude than those spawning at greater depths (e.g., 0.6–0.7 m). Finally, shifts in habitat structure (e.g., submerged macrophytes), thermal regimes (Figure 9), and food web dynamics induced by fluctuations may differentially affect species depending on their trophic niches and microhabitat requirements. By contrast, pelagic spawners with drifting eggs face distinct challenges, primarily related to altered spawning ground locations, drift distances, and downstream incubation conditions, highlighting divergent impact pathways across reproductive guilds.

Accordingly, our habitat model quantifies the direct detrimental effects of drawdown regimes on a key life stage (incubation) for representative adhesive‐egg spawners, while also emphasizing that the severity of these impacts is modulated by species‐specific life‐history strategies (e.g., spawning phenology, egg development rate, oviposition site selection). These species‐specific insights significantly enhance the ecological relevance of our conclusions, underscoring that effective ecological regulation strategies must move beyond generic approaches (Wedekind and Müller 2005). Tailored management, such as fine‐tuning the timing and rate of water level declines to align with the critical reproductive windows and stability requirements of target species or particularly vulnerable guild members, is crucial for conserving the diverse adhesive‐egg fish assemblage within the TGR tributaries. This study provides important insights for developing more effective ecological regulation strategies to support fish habitats in reservoir areas and to inform broader river management efforts in backwater zones.

The uncertainty in this study originates from the input data of habitat factors and the habitat suitability curve (Lin et al. 2015). The uncertainty in the input hydrodynamic data stems from the results of the two‐dimensional hydrodynamic model. The uncertainty of the HSC arises from the selection of hydrodynamic variables in the model and the HSI equation (Boavida et al. 2013). In this study, the accuracy of the simulated velocity of the Pengxi River was 0.91 and 0.89. The HSC was developed based on a recent field investigation of spawning habitats in the Three Gorges Reservoir area, which confirmed the possibility of spawning in the tributaries following the impoundment of the Three Gorges. However, the riverbed substrate was not considered in this study. Although the spatial congruence between optimal depth zones (0.4–0.6 m) and submerged macrophyte distribution (80% overlap) justifies our model's exclusion of explicit substrate representation, heterogeneity in riverbed substrates may still exert secondary influences on habitat suitability (Negro et al. 2021).

Additionally, the two‐dimensional habitat model can only provide average values of hydrological variables and does not capture vertical hydrodynamic variation. This limitation may affect the assessment of egg adhesion success in the following ways: (1) Near‐substrate velocities can be 30%–50% lower than surface flows (Bahmanpouri et al. 2022), potentially allowing eggs to remain adhered even when surface velocities exceed the 0.5 m/s detachment threshold (Figure 2). (2) Surface water temperatures are typically higher than middle‐lower water temperatures. Thus, even if the surface water temperature is higher than the appropriate water temperature range, the middle‐lower water temperature may still be suitable for fish to lay eggs (Figure 4). In future work, a three‐dimensional hydrodynamic model and a suitability index that includes riverbed substrate characteristics should be incorporated to improve the habitat model.

## 5 Conclusion

Our findings demonstrate that the operational scheme of the Three Gorges Reservoir has significantly altered functional habitat connectivity for adhesive‐egg‐producing fish (exemplified by carp and crucian carp) in its tributaries. By developing a habitat model incorporating a five‐day water level decline index, we reveal how dam‐induced fluctuations disrupt the synchronicity between suitable spawning sites and stable incubation conditions. The 39.5% contraction in highly suitable incubation areas and the 34.3% reduction in WUA from spawning to hatching highlight the acute vulnerability of the incubation stage under reservoir operations, posing a serious threat to species that rely on stable hydraulic conditions for egg adhesion and development.

Although this threat is inherent to the adhesive‐egg reproductive strategy, our analysis indicates that the magnitude of impact will vary among species within this guild due to differences in key life‐history traits such as spawning phenology, egg development duration, and oviposition microhabitat preference. The remaining suitable habitats function as refugia, but their reduced carrying capacity suggests diminished population resilience.

These changes underscore the critical importance of integrating hydrological management with ecological considerations, particularly species‐specific requirements, to mitigate the effects of reservoir operations on reproductive success and ensure long‐term species sustainability. This study provides valuable insights for developing more effective, targeted ecological regulation strategies to support diverse fish communities in reservoir areas and informs broader river management efforts in backwater zones affected by dams.

## Author Contributions

**Chenguang Xiang:** data curation (equal), formal analysis (equal). **Wei Huang:** funding acquisition (equal), project administration (equal). **Cuixia Yao:** data curation (equal), project administration (equal). **Huaidong Zhou:** methodology (equal), supervision (equal). **Zhuowei Wang:** data curation (equal), investigation (equal). **Jing Wang:** data curation (equal), investigation (equal). **Pan Yang:** data curation (equal).

## Conflicts of Interest

The authors declare no conflicts of interest.

## Supporting information


**Data S1:** Supporting Information.

## Data Availability

The data can be found in the Supporting Information–S1.
